# Polydimethylsiloxane gel thickness and stiffness affect the initial adhesion of *Escherichia coli* and *Staphylococcus aureus*

**DOI:** 10.1039/d5lp00227c

**Published:** 2025-11-28

**Authors:** Brandon Barajas, Meng-Chen Chiang, Dylan Lechner, Uzochi Uwazuruonye-Anyanwu, Jessica D. Schiffman

**Affiliations:** a Department of Chemical and Biomolecular Engineering, University of Massachusetts Amherst Amherst Massachusetts 01003-9303 USA schiffman@umass.edu; b Materials Science and Engineering Graduate Program, University of Massachusetts Amherst Amherst Massachusetts 01003 USA

## Abstract

The persistent presence of hospital-acquired bacterial infections and the growing prevalence of antibiotic-resistant bacterial strains necessitates a greater understanding of the initial adhesion of bacteria to biomaterials. While the mechanical properties of polydimethylsiloxane (PDMS) gels have been shown to influence the initial attachment of microorganisms, to date, attachment has only been assessed on gels that are 1000× larger than the microorganisms evaluated. Here, a library of nine PDMS gels were manufactured to be thin (∼10 µm), medium (∼35 µm) and thick (∼100 µm) with distinct Young's Moduli that were considered to be soft (*E* = ∼60 kPa), standard (*E* = ∼1150 kPa), and stiff (*E* = ∼1700 kPa). All gels were well characterized using atomic force microscopy. Next, the initial adhesion of microorganisms to the gels was assayed using two strains of *Escherichia coli* (K12 MG1655 and CFT073), as well as two strains of *Staphylococcus aureus* (SH1000 and methicillin-resistant *S. aureus*, *i.e.*, MRSA), representing both well-studied and clinically relevant microorganisms. Bacterial adhesion was the greatest on the thinnest, softest PDMS gels, with *S. aureus* SH1000 demonstrating the greatest changes in adhesive behavior in response to gel thinness. These findings suggest that both PDMS gel stiffness and thickness are important factors when considering the initial adhesion of these Gram-negative and Gram-positive microorganisms to hydrophobic biomaterials.

## Abstract 2

We investigated how the thickness and stiffness of polydimethylsiloxane (PDMS) gels impacted the initial adhesion of *E. coli* and *S. aureus*.

## Graphical abstract

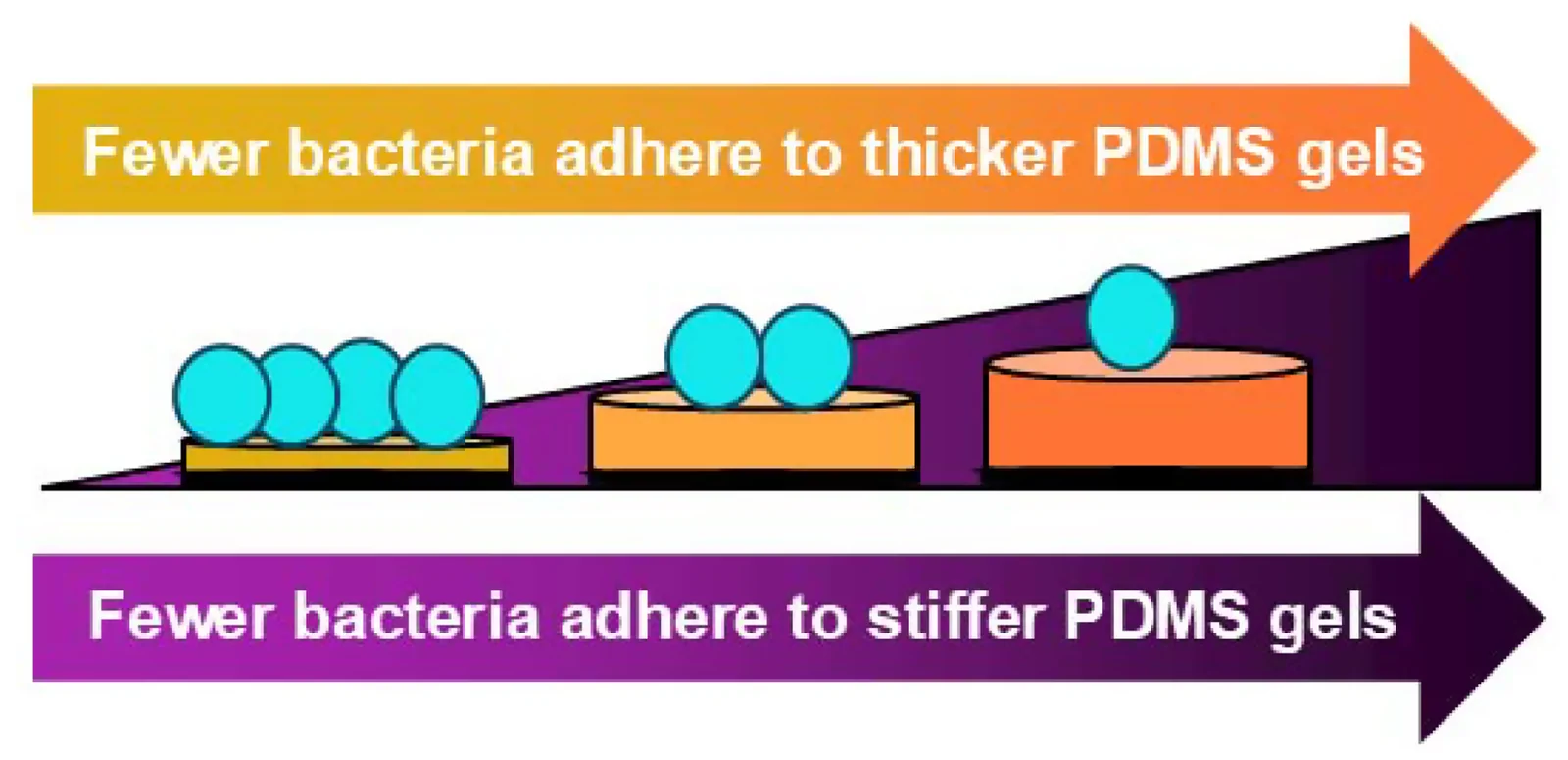

## Introduction

In clinical settings, hospital acquired infections (HAIs) occur when external bacteria enter the body, often through medical devices, which act as common vectors for infection.^1^ Specifically, *Escherichia coli* (*E. coli*) is a major cause of catheter associated urinary tract infections (CAUTIs), whereas *Staphylococcus aureus* (*S. aureus*) is linked to causing central line associated bloodstream infections (CLABSIs).^1^ Catheters most commonly associated with these infections are composed of biocompatible chemistries, such as latex, polyurethane and silicones, with silicone-based catheters being commonly used in both urinary tract and venous settings.^2,3^ Foreign bacteria can attach to these silicone biomaterials and upon insertion into the body, the bacteria can form a biofilm on the device, establishing an infection.^4^ Typically, bacterial infections are treated using commercial antibiotics, however, the repeated use of these antibiotics has led to the evolution of antibiotic-resistant bacteria, such as extended spectrum beta-lactamase (ESBL) producing *E. coli*^5^ and methicillin-resistant *S. aureus* (MRSA).^6^ Other novel antibiotic technologies, such as the use of silver compounds or ions adsorbed by, dispersed in or coated on devices, bulk incorporation of biocidal moieties in a polymer device,^7^ contact killing *via* coatings of proteins such as Hydramycin-1,^8^ and antibacterial polyamine quantum dots,^9,10^ are currently being researched as safer ways to kill bacteria without accelerating antibiotic resistance. To date their effectiveness has been explored in research laboratories, but their widespread clinical adoption remains a challenge due to challenges, such as depletion of biocidal agents, loss in biocidal effectiveness over time, coating durability, and the need for *in vivo* testing.^7,8^

Notably, hospitals have implemented specific sanitation and contact guidelines aimed at reducing infections from antibiotic-resistant bacteria which have been effective. For example, the incidence of MRSA infections decreased by 45–68% from 2015 to 2020 as reported by the Centers for Disease Control and Prevention (CDC).^11–16^ However, the effectiveness of these protocols has been limited, as the same data indicates that the reported cases of MRSA incidences stagnated around 280 000 to 300 000 cases per year from 2017–2020. Additionally, cases of MRSA specifically originating in hospital settings during this timeframe steadily increased to ∼50 000 cases per year.^16^ Preliminary data from a 2022 CDC report on COVID-19's impact on HAIs indicates that the overall incidence of MRSA infections and CLABSIs had regressed to pre-2015 levels, suggesting that progress on reducing HAI incidence was stifled by increased strain on clinical environments.^17^ This startling regression indicates that the current commercially available antibacterials and increased awareness of sanitation protocols are insufficient at preventing and treating HAIs, representing a burden that is felt globally.^18^ Thus, alternative strategies to combat antibiotic-resistant HAIs that prevent the initial adhesion of bacteria are needed.

Instead of focusing on how to treat HAIs after they have developed, an alternative approach is to explore strategies that prevent the initial attachment of microbes from attaching to a surface, thus, delaying or preventing a biofilm from forming on polymer medical devices.^7^ This approach should provide clinicians with more time to identify and treat the attached bacteria while using fewer antibiotics in the process. Understanding how the mechanical properties of a biomaterial can influence the initial adhesion of microorganisms is an emerging concept.^19^ To date, studies have focused on investigating how bacteria adhere to hydrophilic biomaterials, such as agarose, polyvinylpyrrolidones, poly(acrylic acid), polyacrylamide, and poly(ethylene glycol) dimethacrylate as a function of their stiffness, whereas hydrophobic biomaterials have received less attention.^20–28^ Polydimethylsiloxane (PDMS) is a hydrophobic biomaterial that is commonly used in medical devices such as catheters, implant coatings, and biosensors.^29–35^ PDMS is manufactured from a Sylgard 184 kit, commercially available and produced by DOW, that contains a base and a curing agent. The manufacturer recommends mixing them at a “standard” 10 : 1 base to curing (B : C) ratio for optimal crosslinking, stability, biocompatibility, and mechanical stability.^36^ Previously, six studies, one of which is from our group, have quantified the adherence of microorganisms to PDMS gels prepared at the recommended standard 10 : 1 ratio, as well as at 5 : 1, 20 : 1 and 40 : 1 B : C ratios, wherein lower crosslinking leads to softer gels.^37–42^ Greater adhesion to softer PDMS gels was observed across multiple bacterial strains including *E. coli* BW25113, *Staphylococcus epidermis* ATCC 155, *Pseudomonas aeruginosa* DSM 1117, and *S. aureus* ATCC 12 600.^37–42^ Our group has previously reported that more *S. aureus* ATCC12600 and *S. aureus* SH1000 adhered to softer PDMS gels.^42^ To date, these bacterial adhesion studies have been assessed on bulk PDMS gels that were 1.5 millimeters thick or greater.^37–42^ We note that the microorganisms of interest are much smaller than these biomaterials; *E. coli* is cylindrical and approximately ∼1–2 µm long,^43^ whereas *S. aureus* is spherical and ∼1 µm in diameter.^44^ Thus, previous studies have focused on PDMS gels that are approximately 1000× as large as bacteria and thinner PDMS gels that are closer to the formfactor used in hospital settings would be important to explore.

The thickness of PDMS gels used in clinical applications are between 0.5 and 2.5 µm when used in a catheter balloon structure or ∼100 µm if used as a coating.^45,46^ Other polymers used in blood vessel catheters, such as polyurethane, nylon, and polyethylene terephthalate (PET), have thinner wall thickness that are between 15 µm and 60 µm.^47^ Thus, investigations into bacterial adhesion on thinner, micrometer scale biomaterials is both needed and pertinent as it best matches the thickness of catheters used clinically. Previously, Kolewe *et al.* reported that more *E. coli* and *S. aureus* adhered to thinner hydrophilic poly(ethylene glycol) dimethacrylate (*M*_n_ = 750 Da) hydrogels, which were surveyed at three distinct thickness (15, 40, and 150 µm).^21^ When Wang *et al.* explored the attachment of *Pseudomonas aeruginosa* PAO1 to agarose and alginate hydrogels that were 5 µm and 150 µm thick, they found that the Gram-negative microbe adhered more to the thinner hydrophilic hydrogels.^48^ However, to the best of our knowledge, no studies have been conducted that systematically assess bacterial adhesion on hydrophobic PDMS gels as a function of their thickness.

**Fig. 1:**
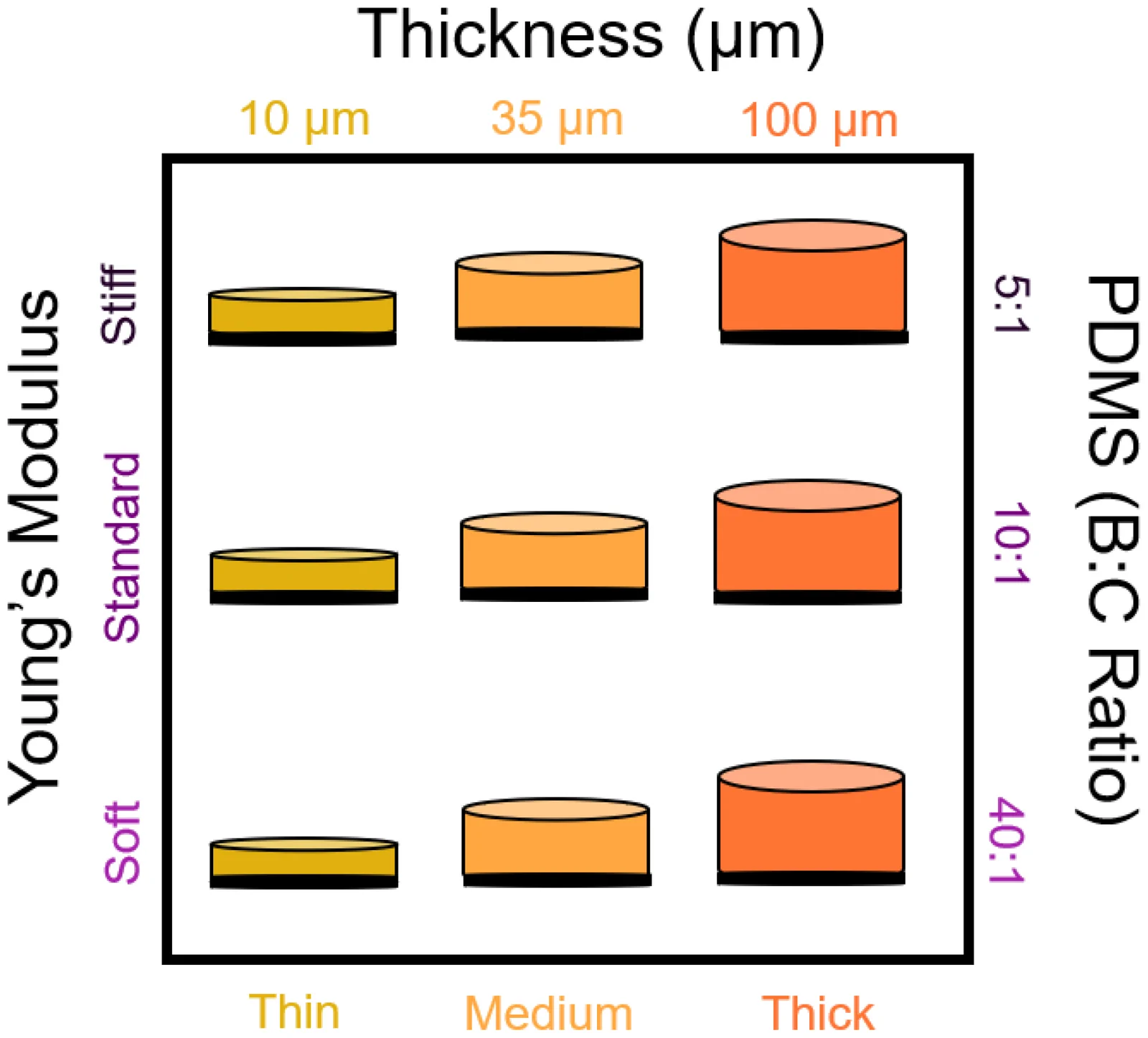
Schematic of the nine PDMS gels evaluated in this study. Throughout the results section, we will refer to a PDMS gel by their thickness and stiffness (bottom and left axes, respectively). The B : C ratio used to fabricate PDMS gels with different stiffnesses is noted on the right axis.

Here, for the first time, we systematically controlled the thickness and stiffness of PDMS gels to decouple their effect on bacterial adhesion. While the previous papers used thick PDMS gels that were cast from viscous solutions, here we used spin-coating to create thin PDMS gels with consistent thicknesses of 10 µm, 35 µm, and 100 µm. Spin-coated PDMS gels have previously been fabricated for applications, such as biomedical nanosystems, superhydrophobic surfaces, flexible bioelectronics, and biomimetic plant leaf surfaces,^49–52^ however, the adhesion of microbes to these gels has not yet been assayed. In this work, by using three distinct B : C ratios of 5 : 1, 10 : 1, and 40 : 1, we also tailored the gel's Young's moduli across three different stiffness regimes. The resulting array of nine spin-coated PDMS gels and their naming schemes are displayed in Fig. 1. The adhesion of well-studied and clinically relevant *E. coli* and *S. aureus* strains were assessed against this array of PDMS gels to determine if the thickness of the gels impacted their attachment. The results of this work provide insights into how medical devices could be designed to reduce the initial attachment of microbes, and hopefully, the incidence of infections that form on hydrophobic PDMS biomaterials.

## Materials and methods

### Materials and chemicals

All compounds were used as received. Sylgard 184 silicone elastomer base and curing agent were purchased from Ellsworth Adhesives (Germantown, WI). M9 minimal salts, d-(+)-glucose, magnesium sulfate, calcium chloride, chloramphenicol (BioReagent grade), glycerol, sodium chloride, yeast extract, tryptone, tryptic soy broth (TSB), propidium iodide (PI), and 4′,6-diamidine-2′-phenylindole dihydrochloride (DAPI) were purchased from Sigma-Aldrich (St. Louis, MO). Deionized (DI) water was obtained from a Barnstead Nanopure Infinity water purification system (Thermo Fisher Scientific, Waltham, MA). OPUS AC-240 (*k* = 2 N m^−1^) atomic force microscopy (AFM) probes were purchased from NanoAndMore (Watsonville, CA).

### Fabrication of spin-coated PDMS gels

PDMS gels with three different Sylgard 184 silicone elastomer base (B) to curing (C) agent mass ratios, *e.g.*, B : C ratios of 40 : 1, 10 : 1, and 5 : 1, corresponding to soft, standard, and stiff gels, were prepared by vigorously mixing together the base and curing agent for 15 min at 20 °C in heavy duty plastic cups (Target Up and Up) that were degassed under vacuum to remove air bubbles.^36^ Gels were spin-coated onto Fisherbrand square microscope cover glass (22 mm × 22 mm) for bacterial assays and circular Fisherbrand glass microscope slides (15 mm diameter) for AFM characterization, which were cleaned by submersion in acetone at 20 °C for 15 min while being gently shaken at 60 rpm. After shaking, the glass slides were rinsed with sterile DI water three times and dried at 70 °C for 16 h in an Oakton StableTemp gravity convection oven before UV/ozone treatment for 15 min using the ProCleanerTM (Bioforce Nanosciences, Ames, IA). Approximately 1 mL of PDMS B : C ratio mixture was dispensed and spin-coated (Laurell Technologies WS-650-23b spin-coater) onto the cleaned slides. Tables 1 and S1 detail the various spin rates explored, from 500 to 4000 rpm. Other parameters were held constant, which include a 2–3 s ramp time and a 60 s hold time. Spin-coated PDMS gels were cured on a hotplate at 60 °C for 16 h in sterile polystyrene Petri dishes (100 × 100 mm, Thermo Fisher Scientific, Waltham, MA).

**Table 1:** Final parameters used to fabricate PDMS gels of distinct thicknesses and stiffnesses. Additional parameters that were explored are provided in Table S1

Stiffness	Thickness	Spin rate^a^ (rpm)	Thickness^b^ (μm)
Soft	Thin	4000	8.6 ± 4.2
Soft	Medium	2300	29.8 ± 9.9
Soft	Thick	1000	101.6 ± 30.1
Standard	Thin	2500	13.2 ± 7.3
Standard	Medium	1750	37.8 ± 9.7
Standard	Thick	1000	95.2 ± 20.5
Stiff	Thin	2300	12.2 ± 6.2
Stiff	Medium	1500	35.4 ± 8.4
Stiff	Thick	700	105.1 ± 16.4

a All samples were prepared using a consistent 2–3 s ramp time and a 60 s hold time.

b Determined using a digital micrometer.

### Characterization of PDMS gels

The thickness of the PDMS gels was determined using a digital micrometer (Mitutoyo Corporation, Kawaski, Japan) by averaging 20 measurements on at least three gels. The hydrophobicity of the PDMS substrates was analyzed using sing a DSA 25S drop shape analyzer (KRUSS Scientific). Static water contact angles were measured using a 2.5 μL droplet of water placed on the substrate. Five separate measurements were performed on three different substrates, probing different portions of the surface with each repetition. Topographical images and local stiffness measurements of the prepared PDMS gels were acquired in duplicate utilizing a Cypher ES AFM (Asylum Research/Oxford Instruments, Goleta, CA) equipped with the 240AC-NA (*k* = 2 N m^−1^, OPUS by MikroMasch, USA) cantilever. Initially, surface topographical images of PDMS gels were acquired in Alternating Current (AC) mode at a scan rate of 0.6 Hz in air. Subsequently, the topographical images were analyzed using Igor Pro (Wave-Metric, Inc., Lake Oswego, OR) to quantify various surface roughness parameters including root mean square roughness (*R*_q_), average roughness (*R*_a_), skewness (*R*_shw_), kurtosis (*R*_kur_), minimum roughness (*R*_min_), and maximum roughness (*R*_max_). The local stiffness of the PDMS gels was assessed using Fast Force Mapping (FFM) mode under a scan rate of 176.06 Hz with the force distance set at 750 nm for 5 : 1 and 10 : 1 and 1.50 µm for 40 : 1 gels. Young's moduli were calculated based on Sneddon's model (see eqn (1)) in Igor Pro due to the conical shape of AFM probe.1
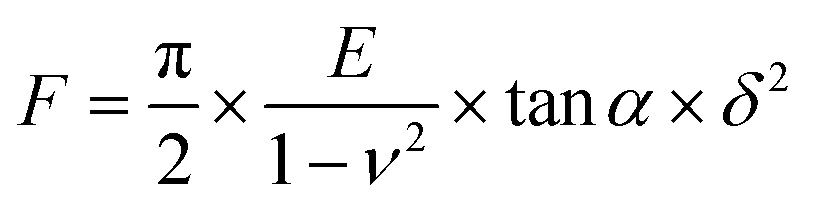
where *F*, *E*, *ν*, α, and *δ* are the applied force, the reduced modulus, the sample's Poisson ratio, the half-opening angle of the AFM tip, and the indentation depth, respectively.^21^

### Bacterial assays on PDMS gels

The Gram-negative strains used include *Escherichia coli* K12 MG1655 (*E. coli* K12), purchased from DSMZ (Leibniz-Institut, Germany) that was transformed with pMF230, a high copy GFP plasmid, as well as *E. coli* CFT073 (a generous donation from Dr Lauren Andrews, University of Massachusetts Amherst). *E. coli* K12 (inoculated with 10 μg mL^−1^ carbenicillin) and *E. coli* CFT073 (inoculated with 10 μg mL^−1^ kanamycin) were cultured overnight in Luria broth media at 37 °C to a concentration of 10^8^ cells mL^−1^. The Gram-positive strains used include *Staphylococcus aureus* SH1000 (*S. aureus* SH1000) with the high efficiency pCM29 sGFP plasmid containing a chloramphenicol antibiotic (a generous donation from Dr Alexander Horswill, University of Colorado Anschutz Medical Campus) and MRSA, which was isolated from a human lung *S. aureus* infection (a generous donation from Dr Robin Patel, Mayo Clinic). *S. aureus* SH1000 (inoculated with 10 μg mL^−1^ chloramphenicol) and MRSA (no antibiotic) were cultured overnight in tryptic soy broth at 37 °C.

Bacterial attachment onto PDMS gels (that were immobilized on glass slides) was assayed using our previously described procedure.^20,21,42^ The PDMS gels and cleaned glass slides (internal controls) were placed in separate wells of 6-well polystyrene plates (Fisher Scientific) before being submerged in 5 mL of M9 medium (11.28 g L^−1^ M9 minimal salts, 20 g L^−1^d-(+)-glucose, 1 g L^−1^ magnesium sulfate, 1 g L^−1^ calcium chloride). Samples were incubated with 10^8^ cells of either *E. coli* K12 (inoculated with 10 μg mL^−1^ carbenicillin), *E. coli* CFT073 (inoculated with 10 µg mL^−1^ of kanamycin), *S. aureus* SH1000 (inoculated with 10 µg mL^−1^ of chloramphenicol) or MRSA (no antibiotic) without shaking at 37 °C for 24 h. All cells were stained with DAPI (10 µM, excitation/emission at 358/461 nm) and allowed to incubate for an additional 10 min and then gently rinsed with M9 medium before acquiring 15 random images per sample using a Zeiss Microscope Axio Imager A2M (20× objective) and Zeiss ZEN 2.3 Pro software. After image acquisition, the chromatogram in the ZEN Pro software was used to remove background fluorescence noise from images prior to further analysis. ImageJ v1.53e was used to calculate the bacteria colony area coverage over the total acquired area, consistent with our previous work.^50^

### Statistical analysis

All bacterial experiments were run in triplicate, and three experiments were conducted per sample type on separate days to account for natural biological variations. Throughout the results, the statistical significance between bacteria area coverage to different gels was determined using the heteroscedastic two-tailed, unpaired Student's *t*-test function using Microsoft Excel Microsoft 365 MSO, Version: 2503 (18.623.20178). Bacterial mean area coverage was reported as the mean (standard error) of three replicates.

## Results and discussion

### Characteristics of spin-coated PDMS gels

Three different silicone elastomer base (B) to curing (C) agent mass ratios (40 : 1, 10 : 1, 5 : 1) were spin-coated to form hydrophobic PDMS gels with different stiffnesses. By systemically controlling the speed of rotation during the spin-coating process, PDMS gels with different thicknesses were successfully manufactured from the viscous precursors, using the parameters provided in Table 1. The spin rates were optimized using previous literature as a starting point^40^ and empirical determination thereafter (see Table S1). A digital micrometer was used to determine that the average thickness of the PDMS gels made from the 5 : 1 B : C ratio was 15.9 ± 1.7 μm, 35.5 ± 8.4 μm, and 109.2 ± 16.3 μm, for the thin, medium, and thick gels, respectively. For the “standard” 10 : 1 PDMS gels, the average thicknesses were 14.6 ± 1.4 μm (thin), 37.9 ± 9.7 μm (medium), and 92.3 ± 20.6 μm (thick). Whereas the gels prepared from the 40 : 1 B : C ratio, had thickness of 9.6 ± 1.3 μm (thin), 29.8 ± 9.9 μm (medium), and 101.6 ± 32.1 μm (thick). All of the PDMS gels were confirmed to be hydrophobic *via* contact angle measurements; the soft PDMS gel had an average contact angle of 120.3 ± 5.6°, whereas the medium was 111.2 ± 2.6°, and the stiff was 110.9 ± 3.2°. These values are consistent with the water contact angles previously reported for bulk PDMS gels.^42^ The full library of nine gels explored in this study are shown schematically in Fig. 1; all thin, medium, and thick PDMS gels had statistically equivalent thicknesses despite being prepared from different B : C ratios, which for simplicity we will round to 10, 35, and 100 μm.

**Fig. 2:**
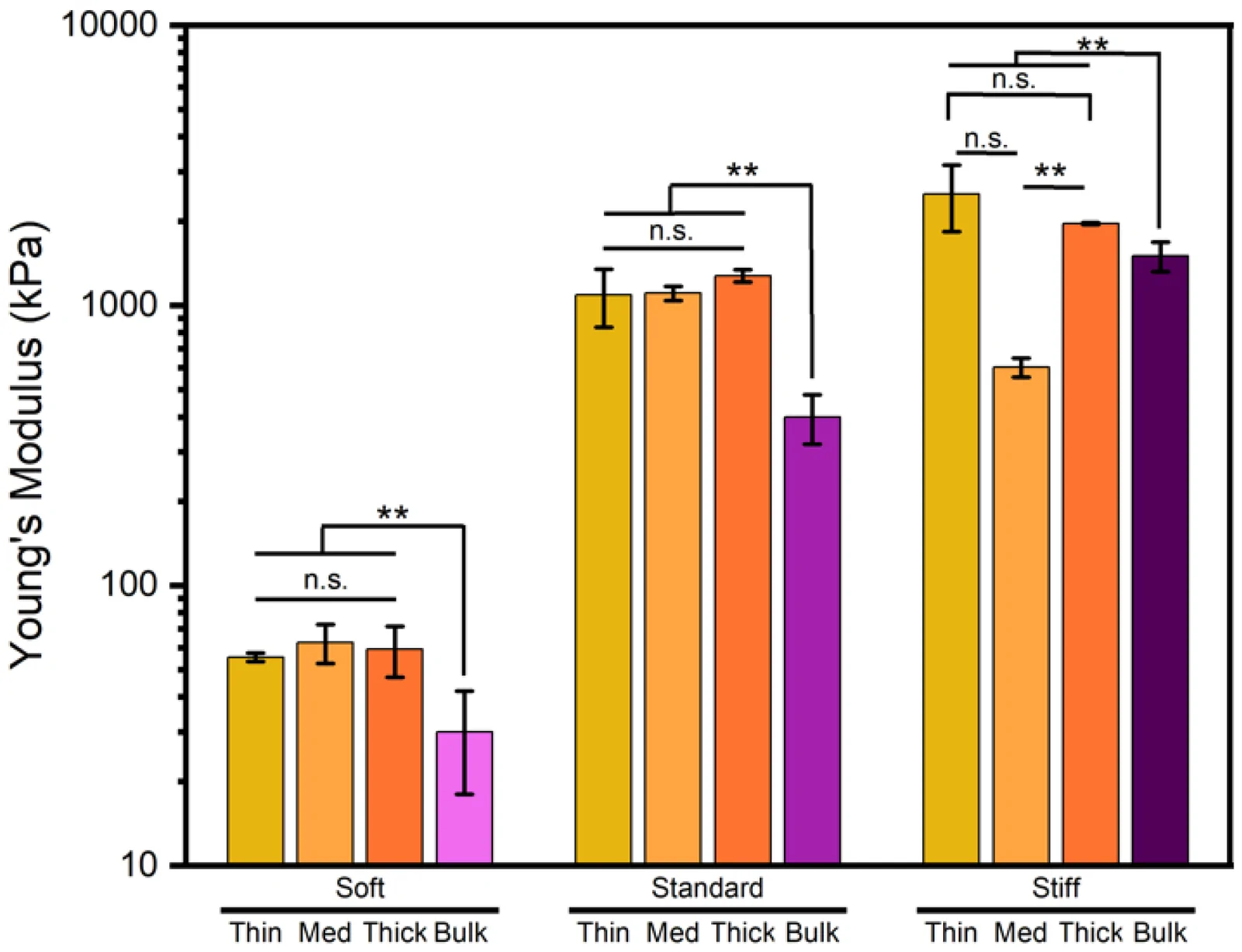
The Young's moduli of spin-coated PDMS gels as a function of their thickness was acquired using FFM AFM. Rheological measurements on “bulk” 5 mm thick gels are also provided.^42^ The error bars denote standard deviation. Two asterisks (**) denote that the values are significantly different at 0.01 level, whereas n.s. indicates that the samples are not statistically different.

As previously mentioned, the mechanical properties of the PDMS gels were tuned by changing the B : C ratios used during spin-coating. The B : C ratios (40 : 1, 10 : 1, 5 : 1) were selected based on our previous work, but because of their thinner geometry, rheological measurements could not be used to confirm their mechanical properties. Thus, fast force mapping (FFM) AFM was used to determine their Young's moduli, as provided in Fig. 2 and Table S2. Representative AFM scans are provided in Fig. S1. The Young's modulus for each sample was within the same order of magnitude for each PDMS B : C ratio, regardless of the thickness of the gel. All “soft” PDMS gels, prepared at 40 : 1 B : C ratio, had statistically equivalent Young's moduli values of 55.5 ± 2, 62.6 ± 9.9, and 59.29 ± 12.2 kPa, for the thin, medium, and thick gels respectively. The standard (10 : 1 PDMS gels) also had statistically equivalent Young's modulus values of 1089 ± 255.2 kPa (thin), 1105.5 ± 63.9 kPa (medium), and 1275.4 ± 63.1 kPa (thick). We note that the only statistically significant difference was between the thin and medium thickness stiff gels; here the stiff gels had a Young's moduli values of 2500 ± 664.7 kPa, 600.9 ± 47.1 kPa, and 1960 ± 14.1 kPa, for the thin, medium, and thick PDMS gels, respectively. In general, the Young's Moduli values acquired using FFM AFM were higher for stiff and standard gels, and similar for the soft gels when compared to rheological measurements acquired on bulk (5 mm thick) PDMS gels.^42^

Regardless of stiffness regime or acquisition method, all Young's modulus measurements were on the same order of magnitude, indicating that the AFM measurements are reliable; they are also comparable to published literature values of AFM measurements on spin-coated PDMS gels. For example, soft PDMS gels have been reported to have Young's moduli ranging from 66 to 206 kPa, standard gels from 1390 to 1480 kPa, and stiff gels from 2400 kPa to 3490 kPa.^51–53^ Overall, the FFM AFM measurements provided assurance that changing thickness of the PDMS gels did not affect the stiffness of the samples.

The average surface roughness of all PDMS gels was very small, and consistently less than 4 nm, as seen in Fig. S2 and Table S3. Representative AFM scans used to quantify the surface roughness parameters are provided in Fig. S3. There was no discernible trend between the thickness of the gels and their root mean square roughness (*R*_q_), average roughness (*R*_a_), skewness (*R*_shw_), kurtosis (*R*_kur_), minimum roughness (*R*_min_), or maximum roughness (*R*_max_). While previous manuscripts have reported that changes in properties like surface roughness, topography, and stiffness can impact bacterial fouling,^51,54^ in this work, the roughness and topography were constant, so we have indeed isolated Young's moduli and sample thickness. Thus, given the low, nanometer-scale roughness, and the size of the *E. coli* and *S. aureus* used in this study (1–2 μm) we hypothesized that surface roughness did not play a major role on adhesion.

### *E. coli* adhesion to spin-coated PDMS gels

**Fig. 3:**
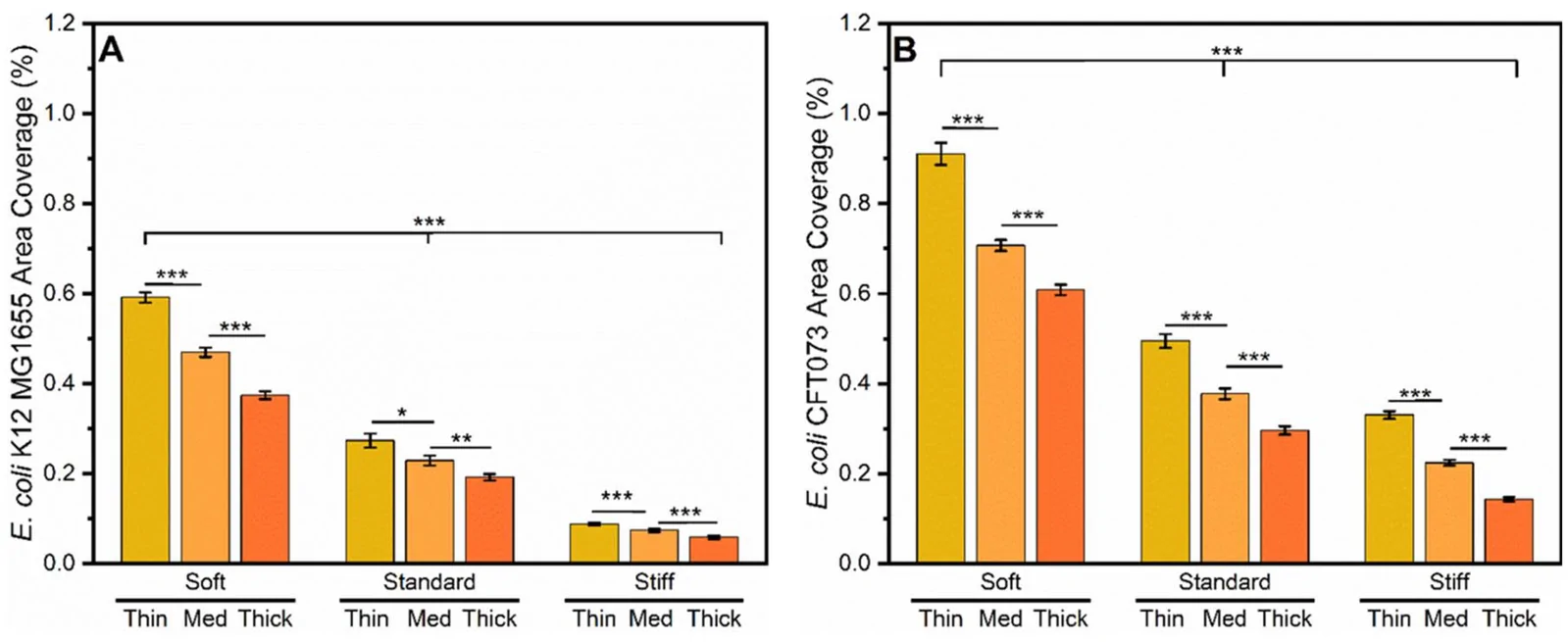
Adhesion of (A) *E. coli* K12MG1655 and (B) *E. coli* CFT073 to spin-coated PDMS gels of various stiffness and thickness after a 24 h incubation period. Standard error is provided. To simplify the plot, the intergroup bar (that compares between different stiffness regimes) are displayed *via* the same bar. One asterisk (*) indicates that the values are significantly different at the 0.05 level, two asterisks (**) indicate that the values are significantly different at the 0.01 level, and three asterisks (***) indicate that the values are significantly different at the 0.005 level.

Next, the PDMS gels of various stiffnesses and thicknesses were exposed to different strains of *E. coli* and *S. aureus* for 24 h. Fig. 3 reveals that both laboratory *E. coli* K12 and the clinical uropathogenic *E. coli* CFT073 strains responded to the PDMS gels in a similar manner. Representative micrographs of *E. coli* adhesion to the gels are provided in Fig. S4. More *E. coli* adhered to softer PDMS gels, which is consistent with previous work using 1.5 mm thick bulk gels.^37,38^ In general, more microbes adhered as the gels became thinner. We note that the total area coverage by the clinical uropathogenic strain was greater than the laboratory strain, highlighting that different strains of the same microbial species display a different magnitude of the same trend.

As calculated using Equation S1 and displayed on Table S4, standard gels tested against *E. coli* K12 MG1655, that were thin, medium and thick had a 53.7%, 51.2% and 48.6% reduced area coverage compared to their counterparts’ soft gels. The thin, medium and thick stiff gels had 85.1%, 84.2% and 84.4% reduced area coverage compared to their counterparts’ soft gels. Changes in the adhesive behavior of the uropathogenic *E. coli* CFT073 strain followed the same trend, as displayed in Fig. 3B and Table S4. For the standard PDMS gels, the thin, medium, and thick had 45.6%, 46.6%, and 50.7% reduced area coverage compared to the equivalent thickness soft gels. Stiff thin, medium, and thick gels had 63.7%, 68.3% and 76.5% less area coverage than the soft gels with equivalent thicknesses. Both *E. coli* strains exhibited similar trends on spin-coated PDMS gels of varying stiffness, but *E. coli* K12 MG1655 appeared to be more sensitive to the effect of stiffness, as the overall reductions in area coverage were greater than those reported for *E. coli* CFT073. While changes in adhesion were similar on both the soft and standard PDMS gels, the difference in area coverage between strains was more pronounced on the stiffer PDMS gels.

A second trend observed in the data is that more *E. coli* adhered to the thinner PDMS gels, as observed on all PDMS stiffnesses, see Fig. 3. To quantify this trend, we calculated the percent reduction with respect to the thin gels using eqn (2); the values are displayed on Table 2.2
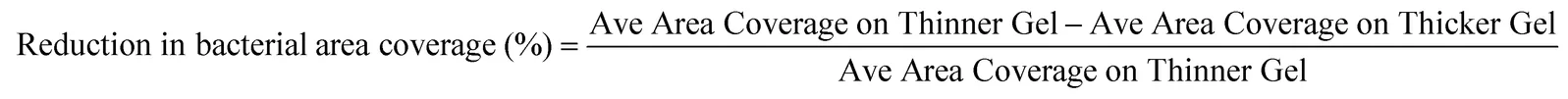


**Table 2:** Change in *E. coli* adhesion as a function of PDMS thickness

*E. coli* strain	Sample	Reduction in bacterial area coverage^a^ (%)	
		Thin to medium	Thin to thick
K12 MG1655	Soft	20.6	36.7
CFT073	Soft	22.3	33.1
K12 MG1655	Standard	16.3	29.7
CFT073	Standard	23.6	40.0
K12 MG1655	Stiff	15.8	33.4
CFT073	Stiff	32.1	56.7

a As calculated using eqn (2).

The omnipresence of this trend across PDMS gels with different stiffnesses suggests that PDMS thickness impacts bacterial adhesion. This observation aligns with results reported by Kolewe *et al.* wherein greater *E. coli* K12 MG1655 adhesion was observed on thinner poly(ethylene glycol) dimethacrylate (PEGDMA) hydrogels.^21^ In that work, PEGDMA hydrogels were spin-coated onto glass coverslips with similar thicknesses (∼15, ∼50, ∼155 µm). The proposed hypothesis for the observed trend was that the bacteria sensed through the thin hydrogels to the glass substrate underneath, which activated surface sensing mechanisms. In the current work, the same bacterial strain along with a uropathogenic variant of the same species again demonstrated increased adhesion to thinner gels; but in this case the gels were hydrophobic PDMS. Both the PEGDMA hydrogels in the Kolewe study and the spin-coated PDMS gels in this study were immobilized on glass substrates. Based on the results of more *E. coli* adhering to thinner PDMS gels, it suggests that similar to the thinner PEGDMA hydrogels, *E. coli* can sense through the thinner PDMS gels, which causes them to respond to the very stiff glass substrate.

Despite PDMS gel thickness affecting bacterial adhesion, its influence on bacterial adhesion appears to be less important than the influence of gel stiffness, as indicated by the magnitude of the differences in area coverage between samples. Across the response of both bacterial strains to PDMS gels of all stiffnesses and thicknesses, changes in material stiffness caused a 46% to an 85% reduction in bacterial adhesion, while changes in material thickness caused a reduced bacterial adhesion, from 16% to 57%. Unlike the area coverage changes associated with gel stiffness, increasing PDMS thickness caused greater decreases in the area coverage of *E. coli* CFT073 than *E. coli* K12MG1655. These observations show that there are differences in bacterial sensitivity to PDMS gel stiffness and thickness and suggest that each *E. coli* strain may have a nuanced response to different materials properties.

### *S. aureus* adhesion to spin-coated PDMS gels

**Fig. 4:**
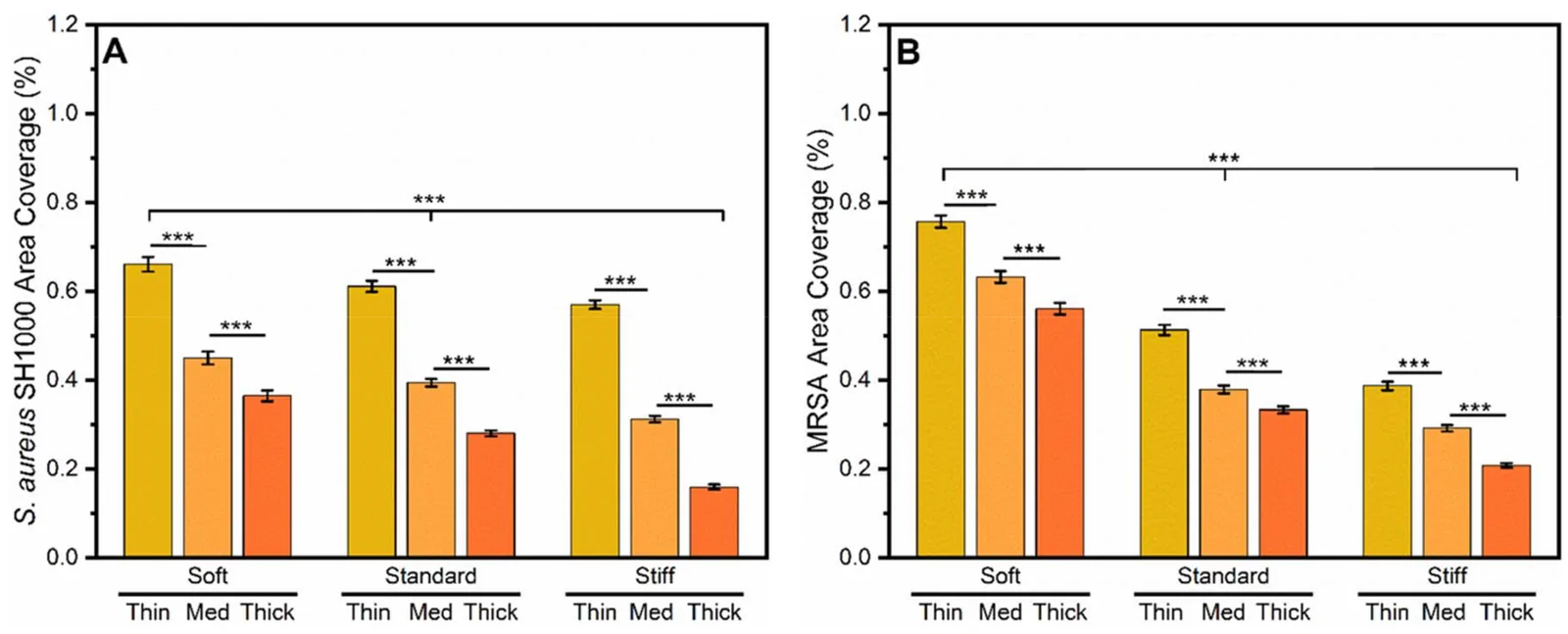
Adhesion of (A) *S. aureus* SH1000 and (B) Methicillin resistant *S. aureus* (MRSA) to spin-coated PDMS gels of various stiffness and thickness after a 24 h incubation period. Standard error is provided. To simplify the plot, the intergroup bar (that compares between different stiffness regimes) are displayed *via* the same bar. Three asterisks (***) indicate that the values are significantly different at the 0.005 level.

Next, the adhesion of the Gram-positive *S. aureus* SH1000 and MRSA strains were assessed using the gel library. Representative micrographs of *S. aureus* adhesion to the PDMS gels are provided in Fig. S5. Again, more *S. aureus* adhered to softer PDMS gels, which is consistent with previous work using bulk gels.^37^Fig. 4A and Table S5 displays that *S. aureus* SH1000, had 7.5%, 12.5% and 23.2% lower area coverage on the stiff *versus* the soft PDMS gels. The thin, medium, and thick stiff gels had 13.7%, 30.6% and 56.3% lower area coverage compared to the soft PDMS gels with the same thicknesses.

The stiffness-associated reduction in area coverage was more pronounced with MRSA than *S. aureus* SH1000. For the standard PDMS gels, the thin, medium, and thick gels had a reduced area coverage of 34.2%, 40.1%, and 40.6% *versus* the soft gels (see trends on Fig. 4B and calculated data on Table S5). For gels in the stiff stiffness regime, the thin, medium and thick gels had 48.8%, 53.9%, and 62.9% reduced area coverage compared to their counterparts in the soft stiffness regime. The response of MRSA to changes in PDMS gel stiffness is more consistent than that of S*. aureus* SH1000, and in most cases also much greater in magnitude. The attachment trends of both *S. aureus* strains followed the same trends that we reported for *E. coli.*

When we closely examine the response of the *S. aureus* strains to PDMS gel thickness we observe that for MRSA, increases in area coverage are present across all gel thickness in each stiffness regime. The data for *S. aureus* SH1000 follows the same trend. Notably, there is a greater area coverage of MRSA than SH1000 on the thin gels, as reported in Table 3. *S. aureus* adhesion was impacted to varying degrees by both the stiffness and thickness of PDMS gels. For MRSA, changes to PDMS stiffness caused a greater reduction of adhesion *versus* changes in thickness, while for *S. aureus* SH1000, changes to thickness caused a greater reduction in adhesion *versus* stiffness.

**Table 3:** Change in *S. aureus* adhesion as a function of PDMS thickness

*S. aureus* strain	PDMS stiffness	Reduction in bacterial area coverage^a^ (%)	
		Thin to medium	Thin to thick
SH1000	Soft	31.9	44.8
MRSA	Soft	16.4	25.9
SH1000	Standard	35.5	54.1
MRSA	Standard	26.1	35.1
SH1000	Stiff	45.2	71.9
MRSA	Stiff	24.6	46.2

a As calculated using eqn (2).

The data trends that resulted from these experiments which were conducted with two different *S. aureus* strains, as with the two different *E. coli* strains, support the conclusion that PDMS thickness impacts bacterial adhesion. Potentially, the bacteria are sensing through the thin gel coatings to the underlying glass substrates. While the surface sensing mechanisms of Gram-positive and Gram-negative microorganisms are different, their trends in adhesion are consistent. Of the four bacterial strains explored in this work, only *S. aureus* SH1000 had a stronger response to PDMS gel thickness, than to stiffness. This suggests that each bacterial strain could potentially display unique responses to different materials properties. Again, we note that the overarching trends of adhesion are consistent, but the degree or magnitude to which they are expressed differs among bacterial strain.

### Discussion on the adhesion of microorganisms to spin-coated PDMS gels

Overall, across multiple gel stiffnesses, and four different bacterial strains with both Gram-positive and Gram-negative bacterial surface structures, more bacteria adhered to thinner PDMS gels. This observation indicates that the effect of PDMS thickness on bacterial adhesion is relevant across bacterial species with differing shapes, cell membrane structures and purported surface sensing mechanisms. Observations in trends of bacterial adhesion as a function of PDMS thickness remained consistent for both rod-shaped and spherical cells, as well as Gram-negative and Gram-positive cell surfaces. The current theory on *E. coli* surface sensing suggests that *E. coli* surface sensing is mediated by appendages on the cell surface such as pili, fimbrae and flagella.^55–57^ When these appendages detect a favorable surface for adhesion, tip adhesin proteins associated with genes, such as fimH and fimA ennable cellular appendages to act as tethers in catch-bond mechanisms to facilitate bacterial attachment. Surface sensing mechanisms of *S. aureus* are much less researched but current literature suggests that adhesion to surfaces is governed by microbial surface components recognizing adhesive matrix molecules (MSCRAMMs), a class of proteins attached to the cell wall of the bacteria that bond to specific ligands.^58^ An example *S. aureus* MSCRAMM is known as clumping factor A (ClfA), which binds to fibrinogen in a catch-bond mechanism that is weak at low tensile force but strong in high physical shear stress conditions.^58^ Even with these completely different biological surface sensing components, the adhesion of these distinct bacterial species exhibited the same patterns in response to PDMS gels of varying thickness. Furthermore, in line with previous studies, the adhesion of other microbes also exhibited the same patterns in response to PDMS gels of varying stiffness. For example, Song and Ren reported that more *E. coli* RP437 and *Pseudomonas aeruginosa* PAO1, two Gram-negative bacterial strains, adhered more to softer 1.5 mm thick PDMS gels, when B : C ratios of 5 : 1, 10 : 1, and 40 : 1 were assayed.^37,38^

While biological processes facilitate the bacterial adhesion process, materials properties, such as thickness and stiffness influence this behavior for many bacterial species. Across both bacterial strains, less-studied clinical isolates demonstrated greater adhesive behavior, suggesting the need to focus on mitigating the adhesion of pathogenic strains to medical devices. While this work focused on the initial adhesion of *E. coli* and *S. aureus*, potentially the results reported here are applicable to other microorganisms. However, more experiments would be needed to draw a definitive conclusion. We note that the focus of this report was on the initial adhesion of bacteria to surfaces, meaning that further work is necessary to fully understand how the materials properties of PDMS gels influence the development and formation of microbial communities and biofilms.

## Conclusions

In this work, for the first time, we have assessed the adhesion of microorganisms to PDMS gels as a function of their thickness and stiffness. Glass substrates were coated with thin (∼10 µm), medium (∼35 µm) and thick (∼100 µm) PDMS layers, which were all much thinner than previously assayed bulk gels. AFM measurements confirmed that the hydrophobic PDMS gels prepared from each B : C ratio had a distinct Young's modulus, which was not impacted by altering gel thickness. The adhesion of *E. coli* K12 MG1655, *E. coli* CFT073, *S. aureus* SH1000, and MRSA decreased with increasing PDMS gel stiffness and thickness, meaning that more bacteria adhered to soft, thinner PDMS gels. However, the magnitude of this effect differed across bacterial strains. For example, thin PDMS gels tested using *E. coli* K12 MG1655, that were soft, standard and stiff had a 36.7%, 29.7% and 33.4% reduced area coverage compared to thick PDMS gels. However, thin PDMS gels that were exposed to *E. coli* CFT073, that were soft, standard and stiff had a 33.1%, 40.0% and 56.7% reduced area coverage compared to thick PDMS gels. An even greater change was observed when thin PDMS gels were exposed to *S. aureus*. Thin PDMS gels tested against *S. aureus* SH1000, that were soft, standard and stiff had a 44.8%, 54.1% and 71.9% reduced area coverage compared to thick PDMS gels. PDMS gel thickness did not impact MRSA as much as *S. aureus* SH1000. Thin PDMS gels tested against MRSA, that were soft, standard and stiff had a 25.9%, 35.1% and 46.2% reduced area coverage compared to thick PDMS gels.

Our findings suggest that both material stiffness and thickness impact both *E. coli* and *S. aureus* adhesion to PDMS gels, as more bacteria adhere to softer, thinner PDMS gels. Outside of the general trends, our findings also suggest that different strains of *E. coli* and *S. aureus* have different sensitivities to thickness and stiffness. Overall, we suggest that it is critical for the design of future biomaterials to consider how the independent tuning of material properties can minimize the initial adhesion of pathogenic microorganisms. Conclusions from this work indicate that to create the most fouling resistant PDMS-based medical devices, such as catheters, that the devices should ideally be both stiff and thick, with wall thickness of at least 100 μm.

## Conflicts of interest

There are no conflicts to declare.

## Supplementary Material

LP-004-D5LP00227C-s001

## Data Availability

The data that support the findings of this study are available from the corresponding author upon reasonable request. The supplementary information includes: thickness data, AFM data and representative micrographs, representative fluorescence images, and bacterial adhesion data. See DOI: https://doi.org/10.1039/d5lp00227c.
