# Supplementary material for: Polydimethylsiloxane gel thickness and stiffness affect the initial adhesion of Escherichia coli and Staphylococcus aureus
Source: RSC Appl Polym. 2025 Nov 28;4(1):353–62. doi: 10.1039/d5lp00227c (PMC12679348; doi:10.1039/d5lp00227c)
Supplement: LP-004-D5LP00227C-s001 [file LP-004-D5LP00227C-s001.pdf]

Supplementary Information (SI) for RSC Applied Polymers.
This journal is © The Royal Society of Chemistry 2025

Electronic Supplementary Information (ESI)

Polydimethylsiloxane Gel Thickness and Stiffness Affect the Initial Adhesion of *Escherichia coli* and *Staphylococcus aureus*

Brandon Barajas[1], Meng-Chen Chiang[1], Dylan Lechner[1], Uzochi Uwazuruonye-Anyanwu[1] and Jessica D. Schiffman[1,2]*

[1]Department of Chemical and Biomolecular Engineering, University of Massachusetts Amherst, Amherst, Massachusetts 01003-9303, United States

[2]Materials Science and Engineering Graduate Program, University of Massachusetts Amherst, Amherst, Massachusetts 01003, United States

*Corresponding author: Jessica D. Schiffman; Email: schiffman@umass.edu

Table S1. Various Spin Rates Were Tested to Manufacture PDMS Gels with Distinct Thicknesses. The Color-Coded Sample Names Represent the Gels Used Throughout the Manuscript.

**B:C Ratio**	**Sample Name**	**Spin Rate** (rpm)	**Thickness** (µm)
40:1	Soft-Test 1	900	125.2 ± 29.6
40:1	Soft-Thick	1000	101.6 ± 30.1
40:1	Soft-Test 2	1400	67.2 ± 30
40:1	Soft-Test 3	1750	44.3 ± 10.2
40:1	Soft-Test 4	2000	41.2 ± 10.4
40:1	Soft-Medium	2300	29.8 ± 9.9
40:1	Soft-Test 5	2800	19.6 ± 5.5
40:1	Soft-Test 6	3000	13.9 ± 5.8
40:1	Soft-Thin	4000	8.6 ± 4.3
10:1	Standard-Thick	1000	95.2 ± 20.5
10:1	Standard-Medium	1750	37.8 ± 9.7
10:1	Standard-Thin	2500	13.2 ± 7.3
5:1	Stiff-Thick	700	105.1 ± 16.4
5:1	Stiff-Medium	1500	35.4 ± 8.4
5:1	Stiff- Thin	2300	35.4 ± 8.4

Table S2. Young's Moduli of PDMS Gels.

Sample	Young's Modulus (kPa)			
	**FFM AFM**			**Rheology**[1]
	Thin	Medium	Thick	Bulk Gel
Soft	55.5 ± 2	62.6 ± 9.9	59.29 ± 12.2	30 ± 12
Standard	1089 ± 255.2	1105.5 ± 63.9	1275.4 ± 63.1	400 ± 80
Stiff	2500 ± 664.7	600.9 ± 47.1	1960 ± 14.1	1500 ± 180

[1]Data from: B. Barajas, I. S. Kurtz, A. J. Waldman and J. D. Schiffman, *ACS Appl. Mater. Interfaces*, 2023, 15, 52197–52206.

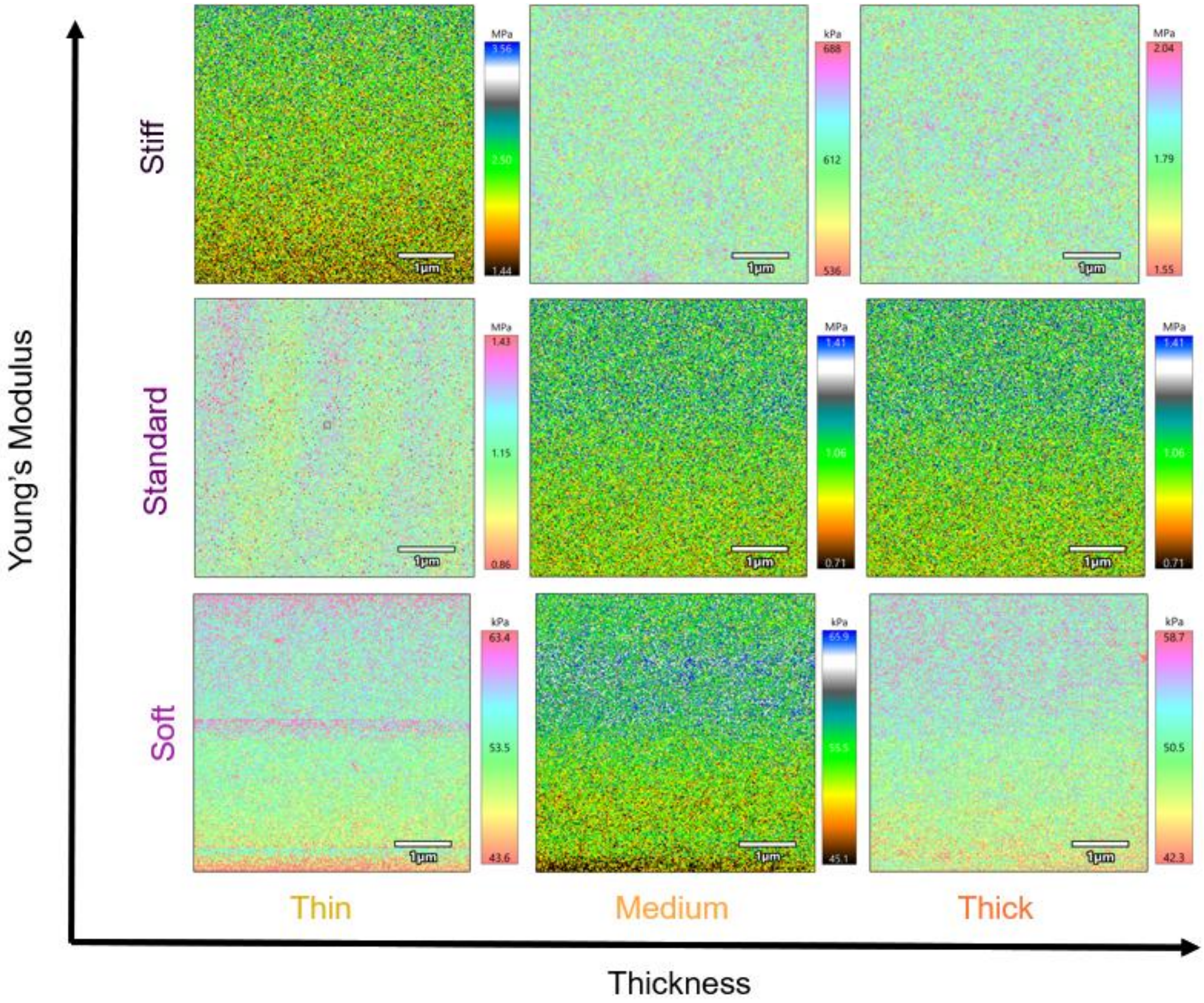

Figure S1. Representative Young's modulus maps acquired on spin-coated PDMS gels. A z-scale is provided alongside each image.

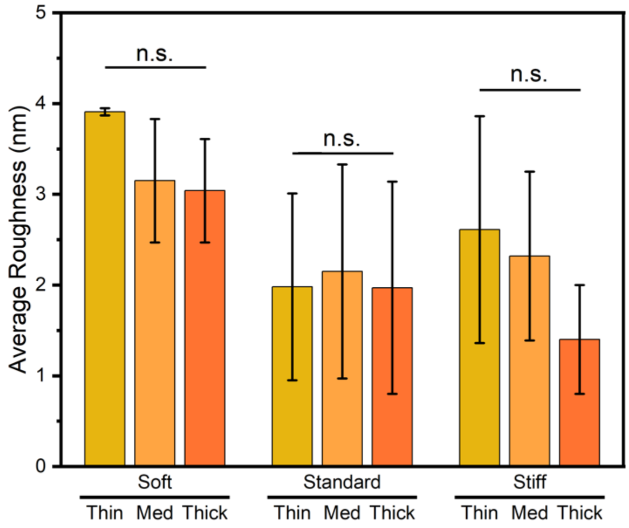

Figure S2. Surface roughness of spin-coated PDMS gels acquired using AFM in AC-tapping mode. Error bars represent standard deviation and n.s. indicates that the samples are not statistically different.

Table S3. Surface Roughness of Spin-coated PDMS Gels Quantified Across 25 μm^2. Provided are the Root Mean Square Roughness (R_q), Average Roughness (R_a), Skewness (R_{shw}), Kurtosis (R_{kur}), Minimum Roughness (R_{min}), and Maximum Roughness (R_{max}). Standard Deviation is Provided.

Sample		R_q (nm)	R_a (nm)	R_{max} (nm)	R_{min} (nm)	R_{skew} (nm)	R_{kurt} (nm)
Soft	Thin	4.92±0.10	3.91±0.04	25.0±17.3	-17.7±1.94	-0.38±0.48	0.22±0.36
	Medium	3.91±0.87	3.15±0.68	10.45±1.19	-14.31±1.92	-0.42±0.30	-0.10±0.27
	Thick	3.77±0.60	3.04±0.57	10.82±2.29	-17.25±1.82	-0.42±0.37	0.18±0.68
Standard	Thin	2.48±1.29	1.98±1.03	20.36±16.87	-10.97±5.36	-0.05±0.08	0.44±0.41
	Medium	2.69±1.48	2.15±1.18	11.0±6.1	-11.31±6.23	-0.004±0.018	-0.001±0.039
	Thick	2.47±1.47	1.97±1.17	11.37±6.94	-12.32±5.74	0.009±0.012	0.11±0.09
Stiff	Thin	3.28±1.57	2.61±1.25	14.38±5.81	-13.83±6.82	0.0698±0.013	0.03±0.01
	Medium	2.91±1.16	2.32±0.93	11.94±5.15	-13.86±5.68	-0.10±0.08	-0.007±0.116
	Thick	1.78±0.76	1.40±0.60	8.03±3.97	-10.99±5.62	-0.25±0.19	0.34±0.22

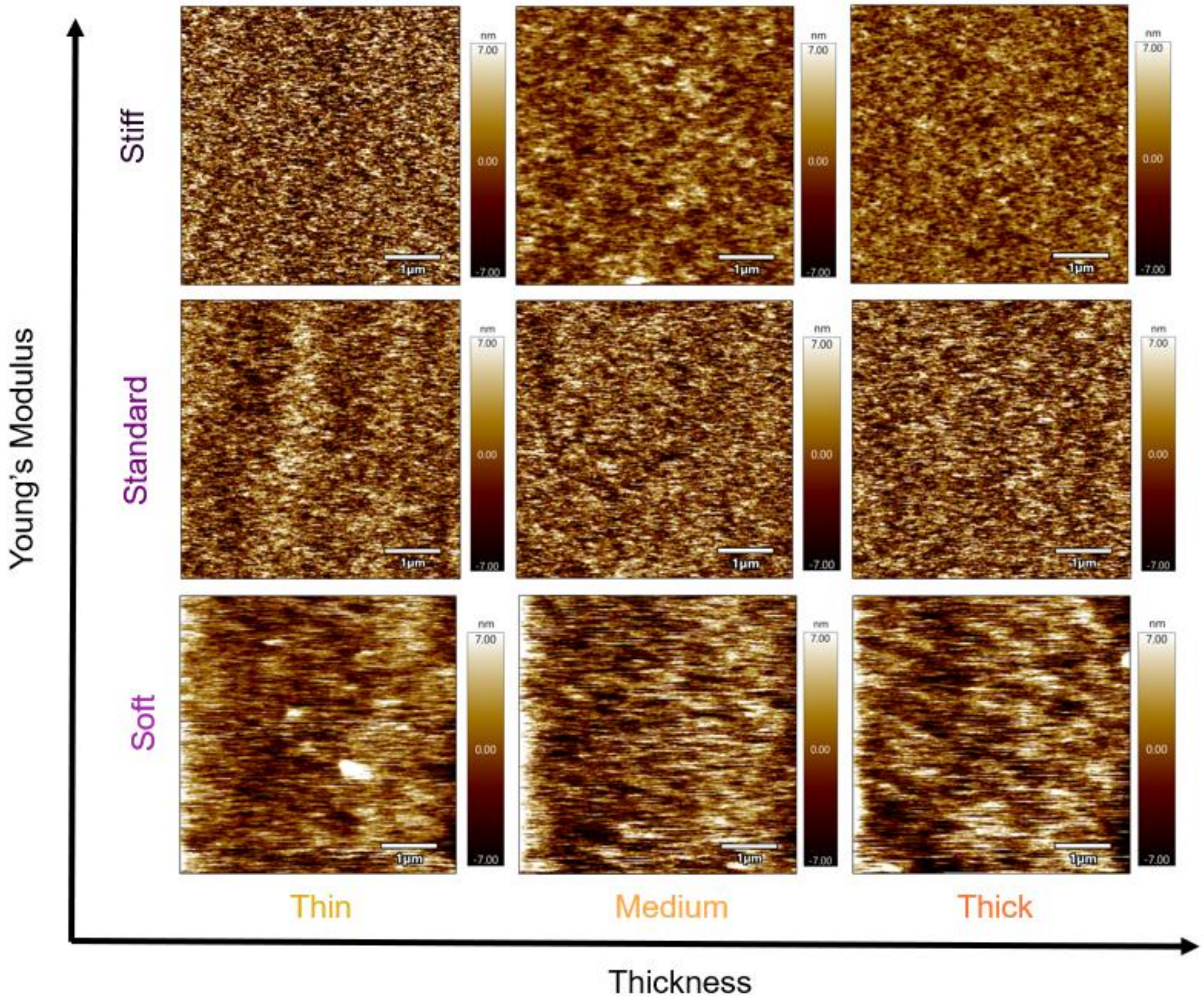

Figure S3. Representative topography images acquired on spin-coated PDMS gels. A z-scale is provided alongside each image.

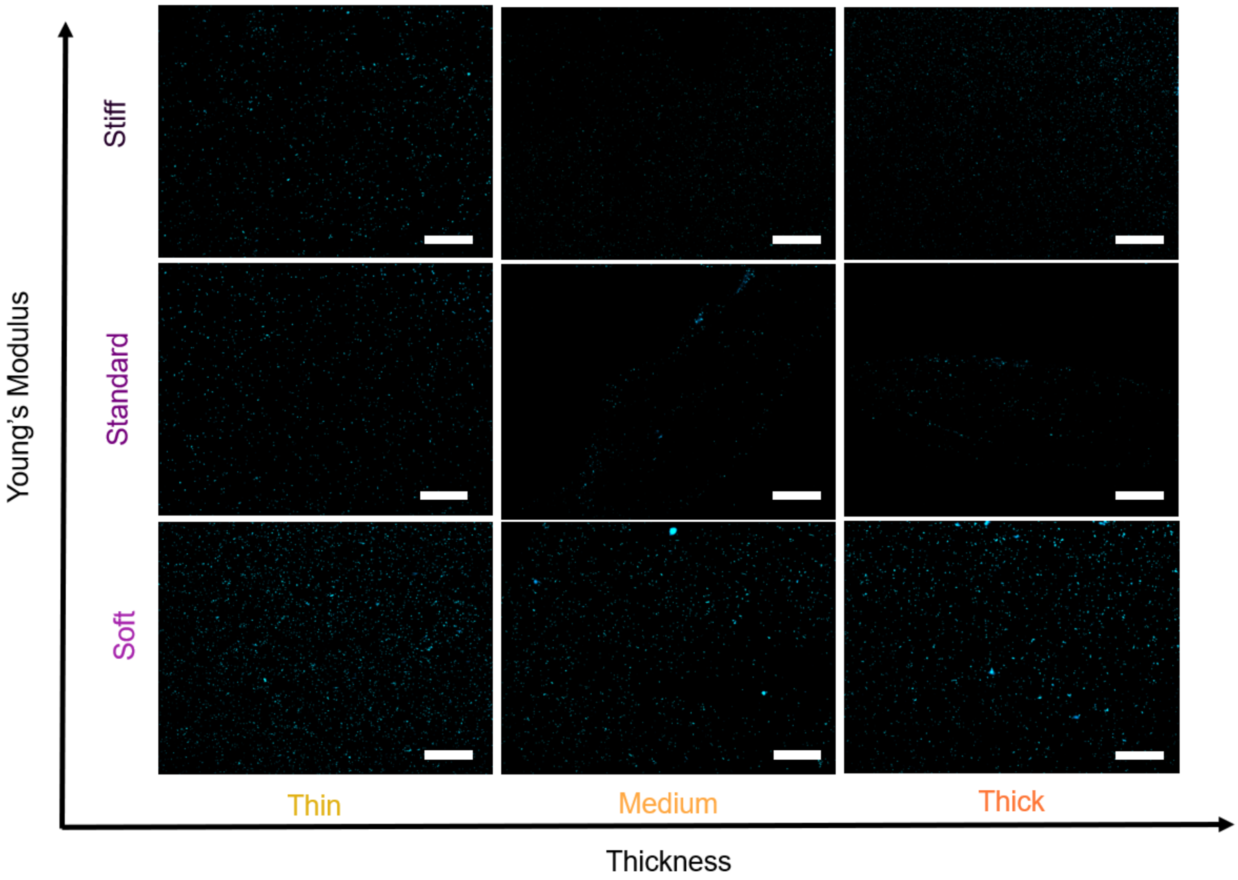

Figure S4. Representative micrographs of *E. coli* CFT073 adhered to various spin-coated PDMS stiffness-thickness combinations. A 100 µm scale bar is displayed.

Equation S1

$$\text{Reduction in Bacterial Area Coverage (\%)} = \frac{\text{Ave Area Coverage on Softer Gel} - \text{Ave Area Coverage on Stiffer Gel}}{\text{Ave Area Coverage on Softer Gel}} \quad \text{(S1)}$$

Table S4. Change in *E. coli* Adhesion as a Function of PDMS Stiffness.

***E. coli* Strain**	**Sample**	**Reduction in Bacterial Area Coverage (%)†**	
		Soft to Standard	Soft to Stiff
K12 MG1655	Thin	53.7	85.1
CFT073	Thin	45.5	63.7
K12 MG1655	Medium	51.2	84.2
CFT073	Medium	46.6	68.3
K12 MG1655	Thick	48.6	84.4
CFT073	Thick	51.3	76.5

†As calculated using **Equation S1.**

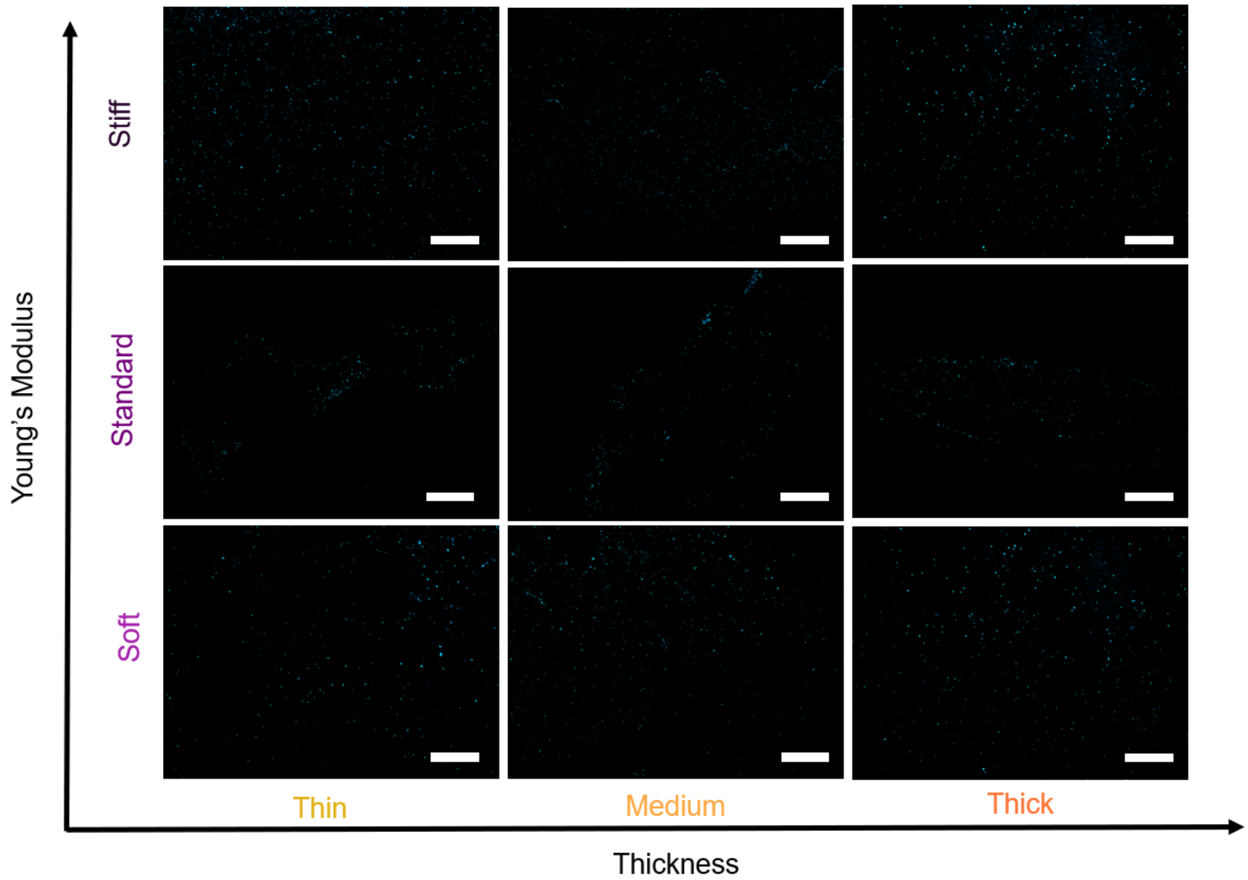

Figure S5. Representative micrographs of MRSA adhered to various spin-coated PDMS stiffness-thickness combinations. A 100 µm scale bar is displayed.

Table S5. Change in *S. aureus* Adhesion as a Function of PDMS Stiffness.

***S. aureus* Strain**	**Sample**	**Reduction in Bacterial Area Coverage (%)†**	
		Soft to Standard	Soft to Stiff
SH1000	Thin	7.5	13.7
MRSA	Thin	32.2	48.8
SH1000	Medium	12.5	30.6
MRSA	Medium	40.1	53.9
SH1000	Thick	23.2	56.3
MRSA	Thick	40.1	62.9

†As calculated using **Equation S1.**
